# Toxicity evaluation of manufactured CeO_2_ nanoparticles before and after alteration: combined physicochemical and whole-genome expression analysis in Caco-2 cells

**DOI:** 10.1186/1471-2164-15-700

**Published:** 2014-08-21

**Authors:** Matthieu Fisichella, Frederic Berenguer, Gerard Steinmetz, Melanie Auffan, Jerome Rose, Odette Prat

**Affiliations:** CEA, IBEB, SBTN, Laboratoire d’Etude des Protéines Cibles, F-30207 Bagnols-sur-Cèze, France; CEREGE, UMR 7330 CNRS/Aix-Marseille Université’, ECCOREV, Europôle de l’Arbois, F-13545 Aix-en-Provence, France; International Consortium for the Environmental Implications of Nanotechnology (iCEINT), Aix-en-Provence, France

**Keywords:** Engineered nanomaterials, Nanoparticles, Transcriptome, Toxicogenomics, Life cycle

## Abstract

**Background:**

Engineered nanomaterials may release nanosized residues, by degradation, throughout their life cycle. These residues may be a threat for living organisms. They may be ingested by humans through food and water. Although the toxicity of pristine CeO_2_ nanoparticles (NPs) has been documented, there is a lack of studies on manufactured nanoparticles, which are often surface modified. Here, we investigated the potential adverse effects of CeO_2_ Nanobyk 3810™ NPs, used in wood care, and their residues, altered by light or acid.

**Results:**

Human intestinal Caco-2 cells were exposed to residues degraded by daylight or in a medium simulating gastric acidity. Size and zeta potential were determined by dynamic light scattering. The surface structure and redox state of cerium were analyzed by transmission electronic microscopy (TEM) and X-ray absorption spectroscopy, respectively. Viability tests were performed in Caco-2 cells exposed to NPs. Cell morphology was imaged with scanning electronic microscopy. Gene expression profiles obtained from cells exposed to NPs before and after their alteration were compared, to highlight differences in cellular functions.

No change in the cerium redox state was observed for altered NPs. All CeO_2_ NPs suspended in the culture medium became microsized. Cytotoxicity tests showed no toxicity after Caco-2 cell exposure to these various NPs up to 170 μg/mL (24 h and 72 h). Nevertheless, a more-sensitive whole-gene-expression study, based on a pathway-driven analysis, highlighted a modification of metabolic activity, especially mitochondrial function, by altered Nanobyk 3810™. The down-regulation of key genes of this pathway was validated by qRT-PCR. Conversely, Nanobyk 3810™ coated with ammonium citrate did not display any adverse effect at the same concentration.

**Conclusion:**

The degraded nanoparticles were more toxic than their coated counterparts. Desorption of the outside layer was the most likely cause of this discrepancy in toxicity. It can be assumed that the safe design of engineered nanoparticles could include robust protective layers conferring on them greater resistance to alteration during their life cycle.

**Electronic supplementary material:**

The online version of this article (doi:10.1186/1471-2164-15-700) contains supplementary material, which is available to authorized users.

## Background

The use of nanoparticles (NPs) has increased significantly during the last decade in several areas such as computer science, chemistry, cosmetics and pharmaceuticals. There is an urgent need to verify their harmlessness in relation to human health and the environment. Cerium oxide (CeO_2_) NPs are one of the most widely used types, for UV protection in paints or as fuel additives [1, 2]. These NPs are usually surface modified to be incorporated into the final commercialized products. Contrary to toxicity studies regarding pristine CeO_2_ NPs, very few studies have focused on manufactured nanoparticles, which are often surface treated to improve their dispersion in liquids. In recent years, CeO_2_ NPs have been shown to induce loss of viability [3] and apoptosis [4] in human lung cells through ROS production, as well as DNA and chromosome damage to human dermal cells [5]. However, these results are controversial. Xia et al. showed a lack of toxicity of CeO_2_ NPs and a protective effect against exogenous ROS [6]. This result is in agreement with the neuroprotective effect of CeO_2_ nanoparticles observed by Schubert et al. [7]. So far, a clear answer to the question as to whether engineered CeO_2_ nanoparticles are toxic or cross biological barriers cannot be provided and more work is required. In addition, these studies focused mainly on inhalation rather than oral exposure, which is also a potential route. To our knowledge, the toxicity of CeO_2_ NPs to intestinal cells was evaluated only recently by B. Gaiser et al. [8]. The authors suggested that both micro- and nanosized CeO_2_ NPs can be taken up by Caco-2 cells, but with little biological consequence, although they suggested that further work would be required to investigate this in more detail. All of these studies focused on the toxicity of pristine CeO_2_ NPs, i.e. at the beginning of the NP’s chain value. However, the NPs that spread in the environment are likely to be degradation residues of CeO_2_-based nanomaterials. Exposure to the environment (UV, water and air contact …) may alter the physicochemical properties of surface-modified CeO_2_ NPs, such as the chemical structure of the surface, size, shape, and dispersion state, which are important parameters for toxicity. After ingestion, stomach acidity can also alter the physicochemical properties of surface-modified CeO_2_ NPs, and their toxic properties. For instance, Wang et al. described an increase in CdSe NP toxicity in intestinal cells after acid treatment, due to degradation of the PEG protective layer [9].

Our study focuses on CeO_2_-based nanomaterials at several stages of the product life cycle. CeO_2_ NPs usually refer to uncoated NPs with UV filter properties. However, CeO_2_ NPs are often formulated prior to use as outdoor paint adjuvants, i.e. the commercialized product, Nanobyk 3810™ (named NB in the text), from the Byk Company. This Nanobyk™ formulation comprises a core of CeO_2_ NPs with a triammonium citrate coating that improves their dispersion in water and paint [10, 11]. The question arises as to whether, during its life cycle, especially by exposure to daylight, this material transforms into a more toxic form for living organisms and the environment.

This work aims to evaluate the relative toxicity of manufactured CeO_2_ Nanobyk™ NPs (NB) compared with their degraded counterparts. Two alteration protocols were used. Firstly, extreme and long-term environmental conditions of aging (100% hygrometry and permanent sunshine) were reproduced, leading to a light-degraded residue (NB-DL). Secondly, gastric degradation was mimicked using a simulated gastric medium, to generate an acid-degraded residue (NB-DA). Surface-untreated (pristine) CeO_2_ NPs from Rhodia were used as a comparative material.

Physicochemical properties (e.g., shape, size, aggregation state, zeta potential and crystal structure) were determined using dynamic light scattering (DLS) coupled to laser Doppler microelectrophoresis for zeta potential measurement, and transmission electron microscopy (TEM). The surface structure and redox state of cerium (Ce^4+^ versus Ce^3+^) were analyzed by X-ray absorption spectroscopy (XAS) [5, 12].

We used the Caco-2 cell line as a human intestinal epithelium model. This cell line has been extensively characterized and shown to exhibit a faithful representation of *in vivo* structural characteristics. At the molecular level, these cells mirror the differentiation of human intestinal cells [13, 14].

We used two viability tests to determine toxic concentrations of these nanoparticles in Caco-2 cells (ATP intracellular measurement and XTT test). Several tests based on different principles are often necessary because NPs may sometimes interact with the test principle [15, 16]. The first cytotoxicity test is one of the most sensitive toxicity assays because it is based on the measurement of ATP, which reflects the energy state of the cell, even before any damage to membrane integrity occurs. The second, and more usual, test (XTT) is based on the activity of mitochondrial enzymes. These tests are routine tests attesting to the presence of dead cells. Nevertheless, some deleterious effects may occur before cell death (inflammation, sensitization, oxidative stress). This is why we also used toxicogenomics, meaning analysis of the whole genomic expression with human pangenomic microarrays to obtain an overview of intracellular events triggered by these various NPs. Additionally, using the same methodology, we also examined the adverse effects elicited by pristine CeO_2_ NPs as comparative material. Hydrogen peroxide was used as positive control, as its effect in Caco-2 cells has been described [17]. We compared the expression profiles of cells exposed to these various particles before and after alteration, using a low concentration (20 μg/mL) about eight times lower than that producing the first visible loss of viability by XTT test. We used scanning electronic microscopy (SEM) to visualize potential adsorption of aggregates onto the cell surface. This multipronged approach gives more certainty and coherence to the acquired data.

## Results

### Physicochemical behavior of (un)altered Nanobyk™ NPs and pristine CeO_2_ NPs

**Figure 1:**
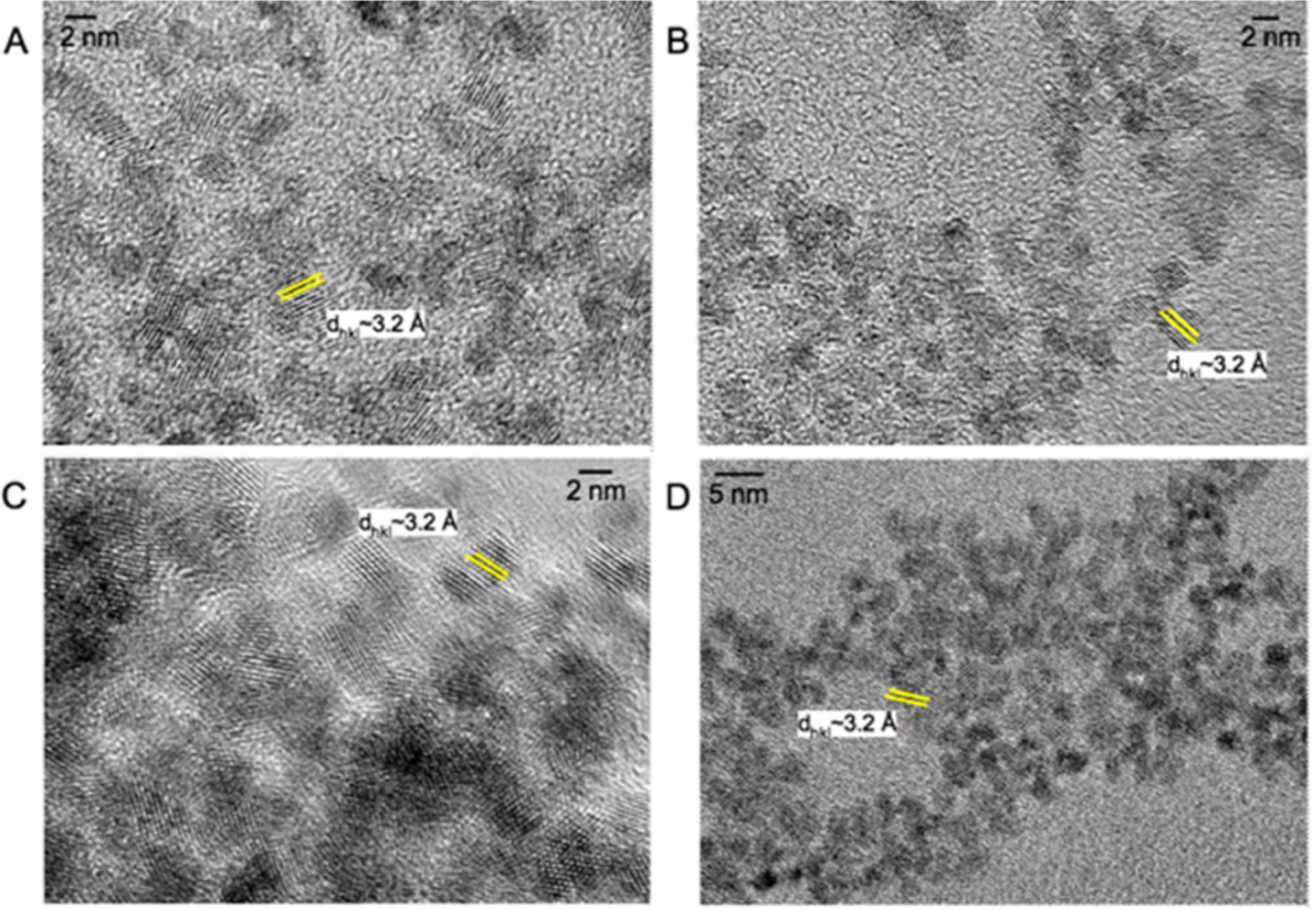
**High-Resolution Transmission Electron Microscope images of CeO**_**2**_ **NPs and Nanobyk™ NPs in deionized water. A)** uncoated CeO_2_ NPs, **B)** NB NPs before treatment, **C)** NB NPs after 4 months aging in daylight (NB-DL), and **D)** NB NPs after acidic degradation (NB-DA). The inter-reticular distance (d_hkl_) measured at ~3.2 Å is attributed to the (111) crystalline plane of CeO_2_. No changes in shape, crystal structure, or CeO_2_ cluster size were observed by HRTEM.

Unaltered Nanobyk™ NPs and pristine CeO_2_ NPs were characterized in pure water. In both cases, TEM showed well-crystallized clusters of cerianite with an inter-reticular distance (d_hkl_) measured at around ~3.2 Å (close to the d_111_ of CeO_2_). These clusters were pseudospherical with diameters of 3 ± 1 nm (average of 50 measurements) (Figure 1). In the stock suspension, the pristine CeO_2_ NPs and unaltered Nanobyk™ NPs were colloidally stable, with average hydrodynamic diameters (Dh) of ~7 nm. At pH ~ 7 ± 0.1, the zeta potential of the Nanobyk™ NPs was negative (−45 ± 5 mV) compared with the positive zeta potential of the pristine CeO_2_ NPs (28 ± 2 mV). After both environmental (light) and gastric (acidic) degradation, the zeta potential of the Nanobyk™ NPs increased (−28 ± 2 mV and −19 ± 2 mV, respectively, at pH 7 ± 0.2). No change in the Ce redox state was observed between the unaltered Nanobyk™_,_ Nanobyk DL and Nanobyk DA (Additional file 1: Figure S1).

The dispersion of NPs has a crucial impact on toxicity. We used DLS to measure the apparent hydrodynamic diameters of NPs in different media. In water, the pristine CeO_2_ NPs and unaltered Nanobyk NPs were dispersed and stable with average hydrodynamic diameters (Dh) of 7 nm. In culture medium, with or without serum, they formed aggregates above 1 μm size. Light- and acid-degraded NPs formed similar aggregates whatever the medium (water, serum-free medium and medium supplemented with 10% FCS). In the presence of 10% serum, in our hands, DLS analyses were especially unreliable, whatever the NPs and concentrations, giving average sizes approaching 2 μm with particle dispersion indexes close to 1. In serum-free medium, the mean hydrodynamic diameters were 1580 ± 1000 nm, 1030 ± 370 nm, 1300 ± 190 nm, and 2200 ± 500 nm for the NB, NB-DL, NB-DA, and the pristine CeO_2_, respectively. It is noteworthy that the samples were not sonicated before any of the toxicity assays.

### Cytotoxicity tests

**Figure 2:**
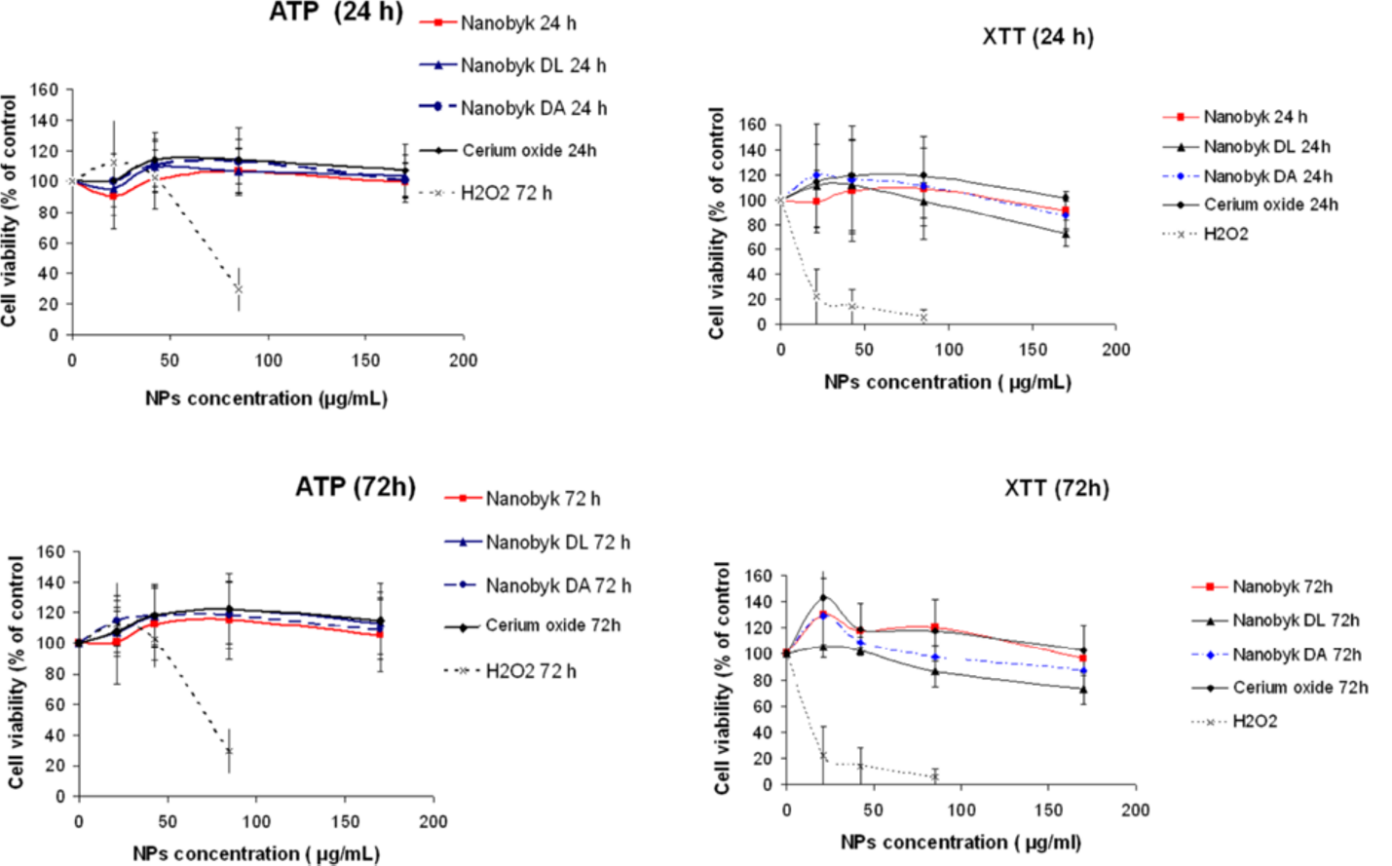
**Caco-2 cell viability tests.** Caco-2 cells were grown in 96-well plates and differentiated for 21 days. Cells were then exposed for 24 h or 72 h to concentrations of CeO_2_ NPs ranging from 21.25 to 170 μg/mL. **Left side)** ATP tests: cell viability was determined by reading the level of bioluminescence (CellTiter-Glo luminescent cell viability assay, Promega). **Right side)** XTT tests: cell viability was determined by mitochondrial enzyme activity via XTT reagent (In Vitro toxicology assay kit XTT based, Sigma-Aldrich). An experimental positive control was obtained by exposing cells to H_2_O_2_ in both tests. Cell viability was not altered for concentrations up to 170 mg/L.

The studies were conducted on the well-established Caco-2 cell line, differentiated for 21 days. The integrity of the cell layer was assessed by measurement of the transepithelial electrical resistance (TEER), stabilized at 500 ohms.cm^2^ after 21 days. We performed two cytotoxicity tests at two exposure times, 24 h and 72 h, to take into account the kinetic parameters. As shown in Figure 2, with the ATP assay (Left side), the presence of CeO_2_ NPs did not induce adverse effects on Caco-2 cells after 24 h or 72 h exposure, even at high concentration (170 μg/mL). The XTT assay (Right side) confirmed the lack of apparent toxicity of Nanobyk NPs and pristine cerium NPs in Caco-2 cells. For degraded Nanobyk NPs (NB-DL and NB-DA) only mild toxicity was observed using the XTT test, and only at the highest concentration (170 μg/mL) after 72 h exposure.

### Cell morphology after exposure

**Figure 3:**
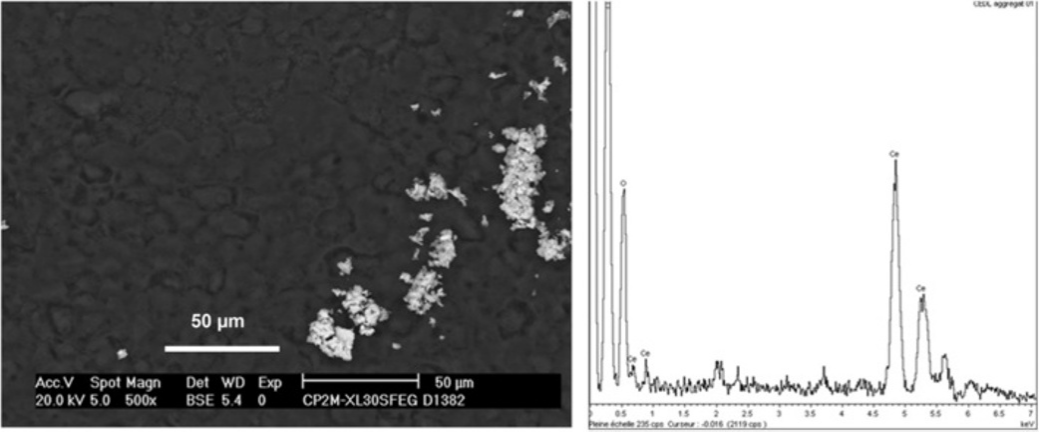
**SEM image and cerium characterization. Left side**: SEM image, obtained in BSE mode, of Caco-2 cells exposed to Nanobyk-DL. Magnification 500 x. **Right side**: The EDX spectrum of the clear spots detected on the cell membrane indicates they are composed of cerium.

We visualized cells with SEM. Caco-2 cells exposed for 72 h to 170 μg/mL Nanobyk-type NPs did not show any alteration in density. The microvilli were also clearly visible (Additional file 2: Figure S2). These observations confirmed the absence of visible toxicity of Nanobyk NPs and indicated that, if an effect exists owing to exposure to degraded NB, this effect is small enough not to induce any drastic changes in cell morphology. It is noteworthy that this does not exclude any possible metabolic modification. Nevertheless, clear spots identified as CeO_2_ deposits were clearly visible on the surface of the plasma membrane, especially for NB-DL (Figure 3).

### Microarray data

Caco-2 cells were exposed to Nanobyk, Nanobyk DL, Nanobyk DA and pristine CeO_2_ for 72 h at 21.25 μg/mL. Cells were also exposed to hydrogen peroxide as a positive control. Agilent Oligo microarrays spotted with 4 x 44,000 probes were hybridized in quadruplicates with RNAs originating from cells exposed to NPs, or from unexposed cells (for exact design details, see Methods). Additional file 3: Figure S3 represents the scatter plots of the raw fluorescence intensities of genes filtered on the threshold intensity signal of the microarray experiments (n = 4). The scatter plot obtained for Nanobyk NPs versus control 1 was very similar to the control scatter plot representing the raw intensities of genes obtained from untreated cells from two different cultures (control 2 versus control 1) giving the false positive rate (5 genes). The exact number of significantly altered genes (fold change ≥ 1.5, p-value <0.05) in each probe set is reported in Table 1. The exhaustive list with gene names and fold changes is shown in Additional file 4: Table S1 under Supporting Information (see Additional file 4).

**Table 1:** **Microarray results**

Microarray Analyses	Number of detected genes	Number of genes up/down- regulated (>1.5 fold change )	Number of genes significantly up/ down- regulated (pvalue < 0.05)	% of genes altered out of detected spots
CTRL 2 vs. CTRL 1	23425	970	5	0.02%
NB vs. CTRL 1	33312	1036	13	0.04%
NB-DL vs. CTRL 1	23962	1773	344	1.44%
NB-DA vs. CTRL 2	22892	2079	428	1.87%
Pristine CeO2 vs. CTRL 3	33023	6020	1643	4.98%
H_2_O_2_ vs. CTRL 3	28900	14651	9307	32.2%

Caco-2 cells were cultured and differentiated for 21 days. The cells were exposed for 72 h to 21.25 μg/mL CeO_2_ NPs, surface-treated or degraded (n = 2). Pristine CeO_2_ NPs at the same concentration and H_2_O_2_ (20 μM) were used as positive controls. After mRNA extraction, labeled cDNA (Cy3) was hybridized (n = 4) to an Agilent oligomicroarray (4 × 44,000 probes). The number of genes detected above the signal threshold was compared for each type of NP versus their own control. From these remaining spots, we selected those with fluorescence ratios (representing NP-treated samples versus untreated samples) above 1.5-fold change. Out of these spots, we selected those satisfying Benjamini-Hochberg multiple testing corrections. At the end of this analysis, we obtained lists of genes that were significantly induced or repressed after exposure to NPs.

Cells exposed to NB showed almost no change in their gene expression, only 13 genes having significantly modified expression. Conversely, cells exposed to degraded NPs had a different scatter plot from the control scatter plot, and had 344 and 428 modified genes for NB-DL and NB-DA, respectively, with 37 common genes listed in Additional file 5: Table S2 under Supporting Information, (see Additional file 5). Cells exposed to pristine (surface-untreated) CeO_2_ NPs displayed much stronger deregulation of their gene expression, with 1643 modified genes. Furthermore, a positive-control scatter plot was obtained with cells exposed to hydrogen peroxide for 24 h at 20 μM (9307 modified genes).

### Biological analysis of the transcriptome

#### Functional analysis

The lists of altered genes were then processed using Ingenuity Pathways Analysis to investigate a possible relationship between altered genes and mechanisms of toxicity.

**Figure 4:**
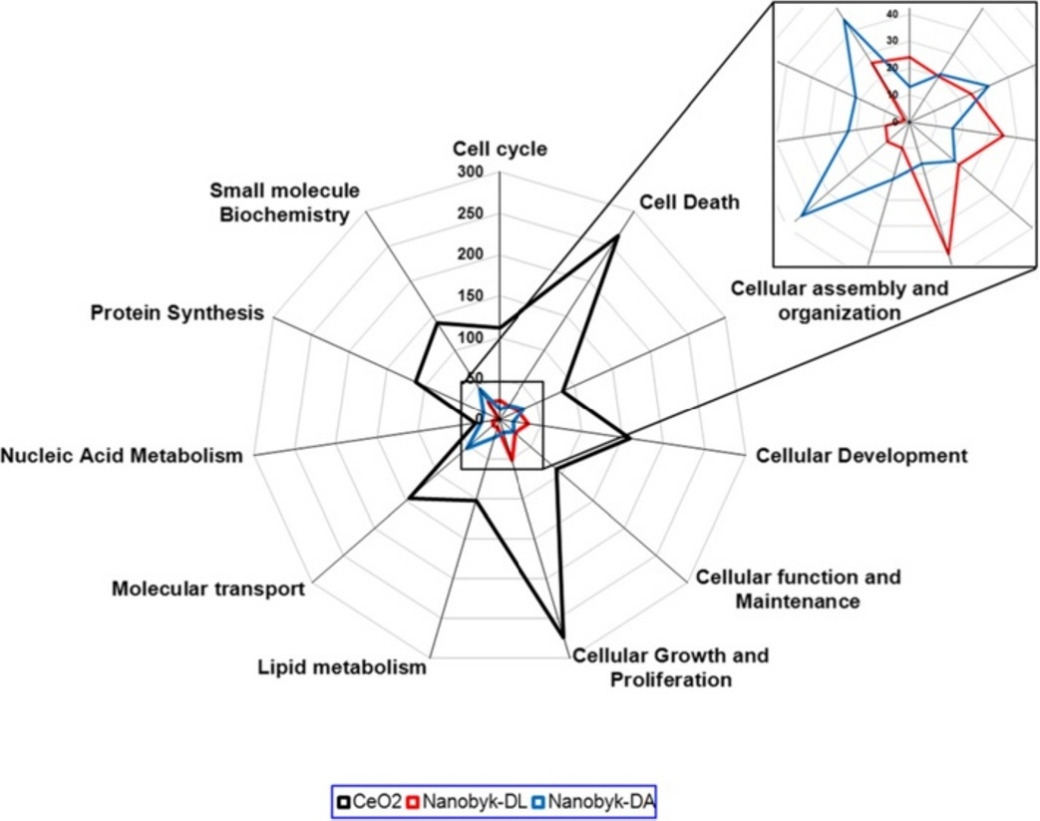
**Radar plots of gene distribution per altered function.** Genes significantly induced or repressed after exposure to NPs were selected as described in Table 1, Column 4. Genes were selected with fold-change ratios greater than 1.5 (n = 4, p-value ≤ 0.05), and distributed per function. This graph displays the number of significantly altered genes per function. The enlarged part concerns light-degraded Nanobyk (NB-DL) and acid-degraded Nanobyk (NB-DA). For each compound, a pattern is obtained representing the amplitude and the nature of its toxicity, then allowing a visual comparison of their respective toxicities.

For pristine CeO_2_ NPs, the main altered functions were cellular growth and proliferation (274 genes) and cell death (265 genes), as shown in Figure 4. Contrarily to pristine CeO_2_ NPs, the surface-treated NB NPs did not alter any specific cellular function (not shown in Figure 4, as only 13 genes are altered), thus proving the efficiency of the protective triammonium citrate layer. The NB nanoparticle residues of degradation by light or acid altered similar functions to pristine CeO_2_ NPs, although in a very moderate way, given the number of altered genes. The distribution per function for NB residues is shown in the enlarged part of Figure 4. For NB-DL, the main altered functions were cellular growth and proliferation (51 genes), cellular development (35 genes), small molecule biochemistry (26 genes), cellular assembly and organization (25 genes), cell cycle (24 genes), cellular function and maintenance (24 genes) and cell death (20 genes). Other functions counted for less than 15 genes each.

For NB-DA, the altered functions were molecular transport (53 genes), small molecule biochemistry (45 genes), cellular assembly and organization (32 genes), nucleic acid metabolism (23 genes), cellular function and maintenance, lipid metabolism and protein synthesis (22 genes each) and cell death (21 genes). Other functions counted for less than 15 genes each.

#### Canonical pathways analysis

**Figure 5:**
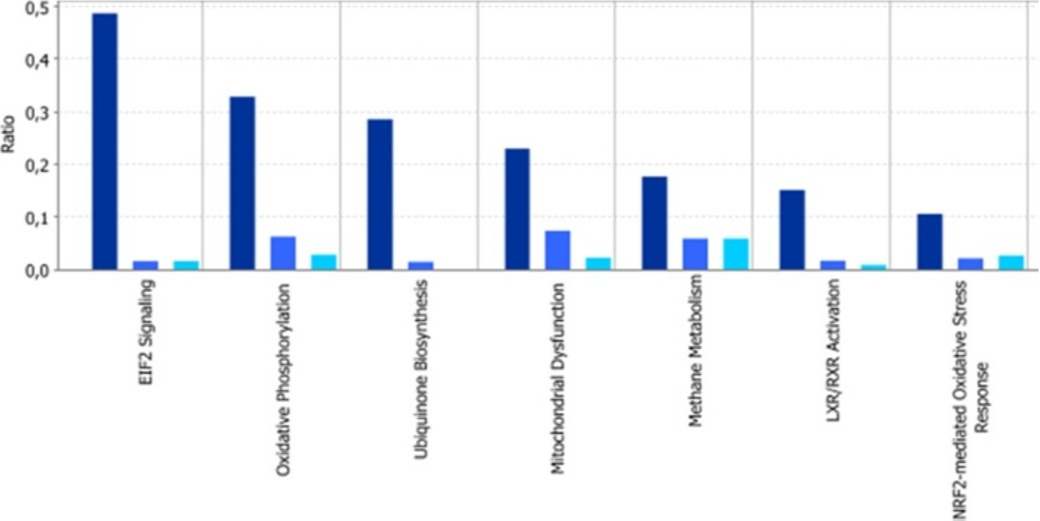
**Comparative analysis of significantly altered pathways by CeO**_**2**_ **NPs (dark blue), NB-DL NPs (medium blue) and NB-DA NPs (light blue).** The y-axis depicts genes ratio within a dataset mapping to the considered pathway (see Methods for calculation). A fisher’s exact test was used to determine a p-value representing the significance of these associations (p < 0.01).

If we look more precisely at the main common molecular pathways definitely impacted by these different NPs (Figure 5), pristine CeO_2_ NPs induced mitochondrial dysfunction through the underexpression of 27 genes of complexes I, III, IV and V, listed in Table 2 Part A. Interestingly, the NB-DA residue also induced mitochondrial dysfunction by underexpressing 10 genes of the complexes I, II, III, IV and VI, listed in Table 2 Part B. Nevertheless, in the case of NB-DA this pathway was affected to a lesser extent. The same trend was observed with NB-DL, which downregulated 3 genes in this pathway, reported in Table 2 Part C.

**Table 2:** **Lists of genes significantly altered by exposure to pristine CeO**_**2**_ **(Part A), NB-DA (Part B) and NB-DL (Part C), and belonging to the canonical pathway “mitochondrial dysfunction”**

Part A: genes altered by pristine CeO2 (n = 27)				
Symbol	Entrez Gene Name	Fold Change	p-value	ID Agilent
ATP5J	ATP synthase, H + transporting, mitochondrial F0 complex, subunit F6	−1,935	8,67E-03	A_23_P154832
COX4I1	cytochrome c oxidase subunit IV isoform 1	−2,313	4,97E-03	A_23_P141029
COX5B	cytochrome c oxidase subunit Vb	−2,312	4,98E-03	A_23_P51069
COX6A1	cytochrome c oxidase subunit VIa polypeptide 1	−2,312	4,99E-03	A_32_P168247
COX6B1	cytochrome c oxidase subunit VIb polypeptide 1 (ubiquitous)	−2,306	4,87E-03	A_23_P108244
COX6C	cytochrome c oxidase subunit VIc	−2,088	3,08E-03	A_23_P8900
COX7A2	cytochrome c oxidase subunit VIIa polypeptide 2 (liver)	−2,311	4,99E-03	A_23_P81690
COX7C	cytochrome c oxidase subunit VIIc	−2,312	4,99E-03	A_23_P110811
COX8A	cytochrome c oxidase subunit 8A (ubiquitous)	−2,312	5,00E-03	A_23_P52639
CYB5R3	cytochrome b5 reductase 3	−2,312	4,99E-03	A_23_P502224
NDUFA3	NADH dehydrogenase (ubiquinone) 1 alpha subcomplex, 3, 9 kDa	−2,052	4,57E-03	A_23_P208540
NDUFA4	NADH dehydrogenase (ubiquinone) 1 alpha subcomplex, 4, 9 kDa	−2,312	4,98E-03	A_23_P145777
NDUFA6	NADH dehydrogenase (ubiquinone) 1 alpha subcomplex, 6, 14 kDa	−2,037	6,10E-03	A_23_P91769
NDUFA8	NADH dehydrogenase (ubiquinone) 1 alpha subcomplex, 8, 19 kDa	−2,200	4,97E-03	A_23_P43566
NDUFB2	NADH dehydrogenase (ubiquinone) 1 beta subcomplex, 2, 8 kDa	−2,175	2,93E-03	A_24_P406301
NDUFB4	NADH dehydrogenase (ubiquinone) 1 beta subcomplex, 4, 15 kDa	−2,301	4,77E-03	A_23_P69468
NDUFB8	NADH dehydrogenase (ubiquinone) 1 beta subcomplex, 8, 19 kDa	−1,963	4,31E-03	A_24_P346886
NDUFB9	NADH dehydrogenase (ubiquinone) 1 beta subcomplex, 9, 22 kDa	−2,311	4,99E-03	A_23_P157669
NDUFS2	NADH dehydrogenase (ubiquinone) Fe-S protein 2, 49 kDa (NADH-coenzyme Q reductase)	−1,561	5,68E-04	A_23_P149470
NDUFS5	NADH dehydrogenase (ubiquinone) Fe-S protein 5, 15 kDa (NADH-coenzyme Q reductase)	−2,311	4,98E-03	A_23_P10463
NDUFS7	NADH dehydrogenase (ubiquinone) Fe-S protein 7, 20 kDa (NADH-coenzyme Q reductase)	−2,079	3,89E-03	A_23_P165086
NDUFS8	NADH dehydrogenase (ubiquinone) Fe-S protein 8, 23 kDa (NADH-coenzyme Q reductase)	−1,904	7,63E-03	A_23_P86774
NDUFV1	NADH dehydrogenase (ubiquinone) flavoprotein 1, 51 kDa	−1,704	1,08E-02	A_23_P127353
OGDH	oxoglutarate (alpha-ketoglutarate) dehydrogenase (lipoamide)	−1,513	3,02E-03	A_23_P123133
PRDX5	peroxiredoxin 5	−1,946	5,32E-03	A_24_P155378
UQCRFS1	ubiquinol-cytochrome c reductase, Rieske iron-sulfur polypeptide 1	−2,031	3,93E-03	A_23_P153586
UQCRH	ubiquinol-cytochrome c reductase hinge protein	−2,312	4,99E-03	A_32_P54137
**Part B: genes altered by NB-DA (n = 10)**				
Symbol	Entrez Gene Name	Fold Change	p-value	ID Agilent
ATP5B	ATP synthase, H + transporting, mitochondrial F1 complex, beta polypeptide	−1,575	2,48E-02	A_23_P33216
ATP5C1	ATP synthase, H + transporting, mitochondrial F1 complex, gamma polypeptide 1	−1,525	3,84E-02	A_23_P63655
COX6A2	cytochrome c oxidase subunit VIa polypeptide 2	−2,028	4,43E-02	A_23_P401524
COX6B2	cytochrome c oxidase subunit VIb polypeptide 2 (testis)	−1,522	2,45E-02	A_23_P78571
CYCS	cytochrome c, somatic	−1,674	3,09E-02	A_24_P376556
NDUFA5	NADH dehydrogenase (ubiquinone) 1 alpha subcomplex, 5, 13 kDa	−1,815	2,45E-02	A_32_P96641
PRDX3	peroxiredoxin 3	−2,65	2,51E-02	A_23_P63751
PSEN2	presenilin 2 (Alzheimer disease 4)	−1,647	2,98E-02	A_23_P96985
SDHB	succinate dehydrogenase complex, subunit B, iron sulfur (Ip)	−1,646	2,46E-02	A_23_P149649
SDHD	succinate dehydrogenase complex, subunit D, integral membrane protein	−1,841	2,39E-02	A_23_P138967
**Part C: genes altered by NB-DL (n = 3)**				
Symbol	Entrez Gene Name	Fold Change	p-value	ID Agilent
COX11	COX11 cytochrome c oxidase assembly homolog (yeast)	−1,723	3,28E-02	A_24_P404204
SDHC	succinate dehydrogenase complex, subunit C, integral membrane protein, 15 kDa	−2,264	3,53E-02	A_24_P233850
SDHD	succinate dehydrogenase complex, subunit D, integral membrane protein	−2,03	4,62E-02	A_32_P60185

Although the same pathway is activated, there are, *stricto sensu*, no common genes altered by pristine CeO_2_ and NB-DA or NB-DL in the mitochondrial dysfunction pathway. This demonstrates the interest of a pathway-driven analysis compared to the usual gene-driven analysis.

#### qRT-PCR validation of the microarray data focused on mitochondrial dysfunction

Validation of the microarray data was confirmed by quantitative RT-PCR on 8 genes belonging to complexes of mitochondrial respiratory chain. The compared fold changes obtained with microarray and with qRT-PCR were reported in Table 3. All mRNAs were downregulated with both techniques, with a good level of significance (p < 0.05).

**Table 3:** **Microarray gene expression validation**

Gene ID	Primers (5′-3′)	Microarray Fold change CeO _2_/Ctrl	qRT-PCR Fold Change CeO _2_/Ctrl
*ATP5J*	F:GTCAGCCGTCTCAGTCCATT	−1.94	−1.75
	R:AAAAGCTCCCTCTCCAGCTC		
*NDUFA4*	F:TCCAGATGTTTGTTGGGACA	−2.31	−5.52
	R:GTGGAAAATTGTGCGGATGT		
*NDUFS7*	F:CGCAAGGTCTACGACCAGAT	−1.68	−2.39
	R:TCCCGCTTGATCTTCCTCT		
*PRDX5*	F:GTGGTGGCCTGTCTGAGTGT	−1.95	−1.74
	R:ATGCCATCCTGTACCACCAT		
**Gene ID**	**Primers (5′-3′)**	**Microarray Fold change NB-DA / Ctrl**	**qRT-PCR Fold Change NB-DA / Ctrl**
*PRDX3*	F:GTTGTCGCAGTCTCAGTGGA R:GACGCTCAAATGCTTGATGA	−2.65	−2.98
*SDHD*	F:GTATGCCTCTTTGCCTCTGC R:GAGGCAACCCCATTAACTCA	−1.84	−4.45
*COX6A2*	F:CTACCAACACCTCCGCATC R:TCGAAGCTTCACACCTTTATTG	−2.03	−2.08
**Gene ID**	**Primers (5′-3′)**	**Microarray Fold change NB-DL / Ctrl**	**qRT-PCR Fold Change NB-DL / Ctrl**
*SDHC*	F:TTGAGTGCAGGGGTCTCTCT R:AACCAGGACAACCACTCCAG	−2.26	−16.30
*SDHD*	F:GTATGCCTCTTTGCCTCTGC R:GAGGCAACCCCATTAACTCA	−2.03	−22.10

F = forward; R = reverse.

## Discussion

Contrary to the toxicity studies regarding pristine CeO_2_ NPs, very few studies have focused on engineered nanoparticles, which are often surface treated for better dispersion in liquid products. However, these particles can be degraded in the environment during their life cycle. Moreover, although the toxicity of pristine nanoparticles via inhalation is well documented [3, 18, 19], only a few evaluations of toxicity associated with oral exposure have been carried out [8]. Here, we investigated the potential toxic effects on the intestine of Nanobyk 3810™ NPs, used as long-term UV protection for wood, and their degradation residues, as compared with pristine CeO_2_ NPs. We considered two degradation scenarios: i) due to environmental conditions, and ii) due to stomach acidity. Caco-2 cells were used for toxicity evaluations and their differentiation was evaluated with the measurement of transepithelial resistance.

Following both environmental or acidic degradation in water, the NB surface becomes less negatively charged at physiological pH than the initial NB (zeta potential −28 ± 2 mV and −19 ± 2 mV for NB-DL and NB-DA, respectively, compared to −45 mV for NB). These negative surface charges are attributed to the negatively charged citrate capping at this pH. Indeed, citrate is a tricarboxylic acid with three dissociated protons (pKa_1_ = 3.13, pKa_2_ = 4.76 and pKa_3_ = 6.40) [20]. Consequently, partial desorption of the citrate layer during alteration will decrease the negative charges at the surface of the Nanobyk™, as already described [21]. While the Nanobyk and the pristine CeO_2_ NPs have hydrodynamic diameters of 7 ± 1 nm in a dispersed state, strong aggregation occurs in the culture medium, with and without 10% FCS. Serum proteins usually help the dispersal of the NPs [22], but for very small NPs with hydrodynamic diameters less than 10 nm the bonds to proteins are so weak and labile that most of the NPs exchange quickly, which favors aggregation, as has been described recently in our laboratory by Liu et al. [23]. Using a culture medium supplemented with 10% FCS and containing 60 μg/mL CeO_2_ NPs, NP distribution with the protein population was analyzed by size exclusion chromatography and ICP-MS, which showed that 90% of the CeO_2_ NP population was eluted in the dead volume as homo aggregates. This is consistent with the results obtained by SEM showing adsorbed NP aggregates on the cell membrane. The hydrodynamic diameters of all NPs tested, including NB-DL and NB-DA, in the cell culture medium are microsized, between 1 and 2 μm, with zeta potentials around −15 mV, leading to strong aggregation, and coherent with published data [18, 23, 24].

Through two different toxicity assays (ATP and XTT), Caco-2 showed no loss of viability after exposure to up to 170 μg/mL over 72 h for Nanobyk 3810™ and pristine CeO_2_ NPs. For degraded Nanobyk NPs, a slight effect (27% loss of viability) was observed for the highest concentration (170 μg/mL) after 72 h with the XTT test, but not with the ATP tests, for NB-DL and NB-DA. ATP and XTT tests are not based on the same principle. XTT is directly based on the enzymatic activity of succinate dehydrogenase, essential to mitochondria. We showed that this enzyme was down regulated in our dataset. ATP test measures the total amount of intracellular ATP that is not decreased in a so sensitive way. This explains why the sole XTT test is disturbed by those NPs. This last test tends to indicate that altered Nanobyk NPs induce a slight change in mitochondrial function. It should be also noted that the XTT test, by induction of a soluble formazan species, does not suffer from the possible interference with NPs frequently described with the MTT assay. This known bias is due to the formation of insoluble MTT formazan, which is often entwined with NP fibers or complexes that inhibit its resolution by the solvent [25]. None of the tested nanoparticles induced drastic changes in cellular aspect as depicted in Additional file 2: Figure S2, excepted deposits on cell membrane, suggesting that the effect was probably a modification of the internal metabolic activity. It is possible that some signaling cascades may be triggered by interaction with membrane proteins such as protein-G coupled receptors [26].

We did not notice any evolution of the redox state (i.e. potential reduction of Ce^4+^ to Ce^3+^) of Ce atoms localized at the surface of the NPs after alteration in water. The shape of XANES spectra at the Ce L3-edge, and the position of the edge, are usually easily distinguished for Ce^3+^ and Ce^4+^ reference compounds: one absorption edge at 5729 eV for Ce^3+^ and a double peak at 5733 eV and 5740 eV for Ce^4+^ (Additional file 1: Figure S1). Consequently, XANES, used to detect any slight change in the redox state of CeO_2,_ indicated no Ce^3+^ in our suspensions.

Standard toxicological methods *in vivo* cannot unravel the mechanisms of action of toxicants. Some *in vitro* methods are able to do so, but rarely at sublethal doses. Conversely, toxicogenomics is a methodology capable of detecting potential changes in cellular and molecular functions at low dose, and constitutes an alternative for assessing the toxicity of nanomaterials [27]. In particular, microarray-based transcriptional profiling is a powerful tool for monitoring altered cellular functions and pathways (i.e. oxidative stress, apoptosis, hypoxia, etc.) under the action of toxicants. Gene expression studies, through a large number of altered genes, provide a wealth of information for sketching the intracellular mode of action of toxic substances [28, 29]. These results usually lead to the generation of new hypotheses about the specific toxicity of the substances concerned, then allowing targeted *in vitro* or *in vivo* experiments. The overall number of altered genes is a very good indicator of the level of cellular disturbance induced by a toxic compound.

The cells were exposed to 21.25 μg/mL of various NPs for 72 h, a concentration about eight times lower than that producing the first visible loss of cell viability at 72 h with the XTT test. This exploratory concentration was chosen in the absence of any oral reference dose for cerium oxide nanoparticles. In the current study, toxicogenomic results showed that, by exposure to NanoByk NPs capped with ammonium citrate, Caco-2 cells were almost unaffected, as only 13 genes were altered out of 44,000 transcripts (Table 1). On the contrary, by exposure to pristine CeO_2_ nanoparticles used as the reference material in the same conditions, cells underwent a strong alteration of gene expression, with 1,643 significantly differentially expressed genes. In short, capping nanoparticles provides a very good protection against adverse cellular effects. On the other hand, light- or acid-degraded Nanobyk NPs moderately altered the transcriptome of Caco-2 cells, with 344 and 428 modified genes, respectively, including 37 common genes (Additional file 5: Table S2). These alterations were much less important than those obtained when cells were exposed to pristine CeO_2_ NPs at the same concentration (1,643 genes). For instance, cell death was more highly activated for CeO_2_ NPs (265 genes) than for NB-DL and NB-DA (about 20 genes each). Otherwise, we noted that there was no cytokine production (chemokines or interleukins), indicating an absence of proinflammatory response with all the NPs tested.

To identify the mechanisms involved, we analyzed the distribution of altered genes per function, as defined in Gene Ontology (Figure 4). Radar plots may help here to apprehend the complexity of toxicity, both in terms of amplitude and effect. This results in a specific pattern representing the toxicity of each compound. Graph overlay of several products allows the visual comparison of their respective toxicities. Once again, the magnitude of the effect is given by the number of altered genes in a given function. For NB-DL, as for CeO_2_ NPs, the main altered functions were similar: cellular growth and proliferation (51 vs. 274 genes), cellular development (35 vs. 159 genes), cell death (20 vs. 265 genes) and small molecule biochemistry (26 vs. 140 genes). This may suggest that their modes of toxic action are similar. For NB-DA, the main altered functions related to molecular transport (53 genes) and small molecule biochemistry (45 genes). The functional mechanisms of toxicity were slightly different between the two residues, although this type of analysis is more informative with a higher number of genes. Small molecule biochemistry was an altered function in all cases.

Although the functions provide valuable information on the actions of the involved genes (transport, cell growth, etc. ..), the pathways help in understanding, in a faster and more extensive manner, the interactions between these genes themselves and the cellular mechanisms to which they belong. In the present study, pristine CeO_2_ NPs greatly disturbed the EIF-2 signaling pathway by downregulating the expression of more than 90 ribosomal proteins (S and L type), with a strong overall impact on protein synthesis (Figure 5). This result was consistent with the results of L. Benameur [30], where pristine CeO_2_ NPs triggered intracellular damage, such as the disruption of antioxidant systems (GSH, SOD, GPx, ascorbate), and impacted on protein synthesis in general.

**Figure 6:**
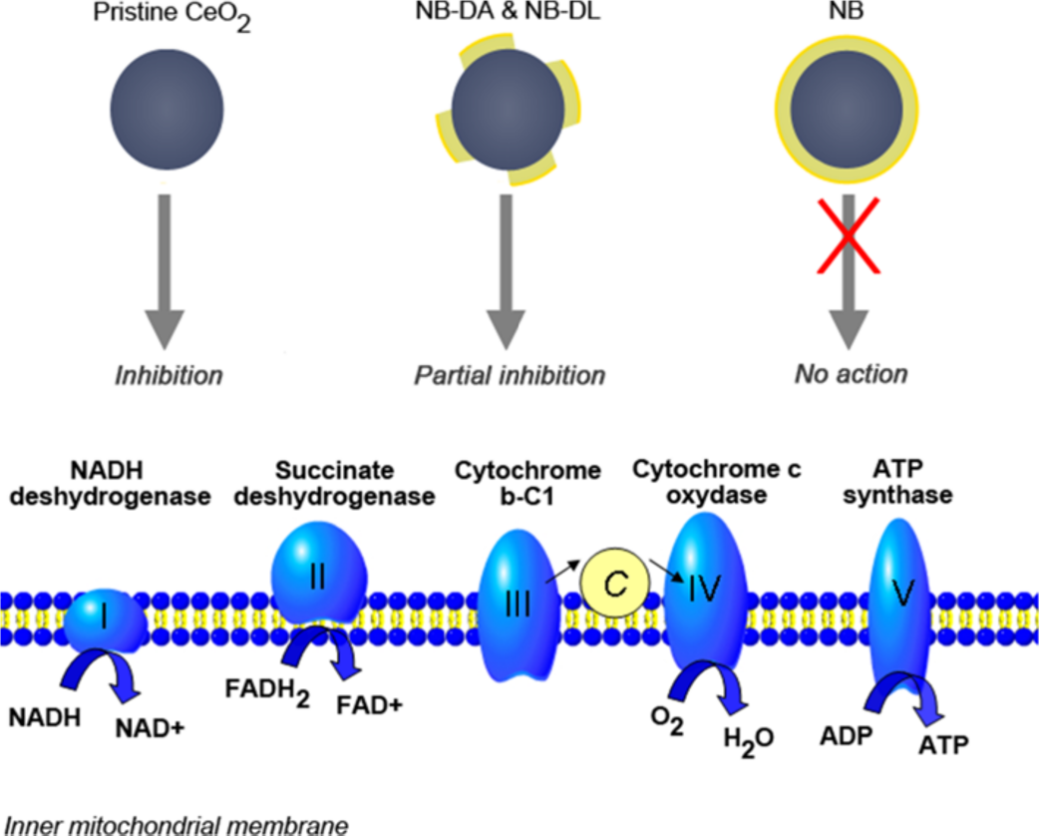
**Complexes of the respiratory chain altered by degraded Nanobyk.** Pristine CeO_2_ under-expressed 27 genes encoding subunits of complex I, III ( cytochrome b-c1), IV and V. Acid-degraded Nanobyk downregulated 10 genes encoding subunits of complex I (NADH dehydrogenase), complex II (succinate dehydrogenase), complex IV (cytochrome c oxidase) and complex V (ATP synthase). Light-degraded Nanobyk down regulated 3 genes encoding subunits of complexes II and IV. Nanobyk NPs did not alter the respiratory chain. Differentially expressed genes belonging to this specific pathway are listed in Table 2.

Comparison of the common canonical pathways disturbed by pristine CeO_2_ NPs and degraded NB NPs essentially showed that they all disturbed mitochondrial functions and the oxidative phosphorylation pathway. Oxidative phosphorylation is the mitochondrial process by which ATP is formed as a result of the transfer of electrons from NADH or FADH2 through a series of electron carriers to oxygen. Consequently, this pathway is modified as a result of mitochondrial dysfunction (Figure 6). The downregulation of some key genes of oxidative phosphorylation was validated by qRT-PCR (Table 3).

This point confirms the results obtained above with the XTT test, which involved the mitochondrial enzymatic process. Moreover, the succinate dehydrogenase complex is clearly underexpressed by NB-DL and NB-DA (*SDHB, SDHC* and *SDHD*) whereas the NADH dehydrogenase complex (*NDUFA, NDUFB and NDUFS*) is downregulated by pristine CeO_2_ NPs. This impairment is, however, visible by toxicogenomics at a concentration divided by 8 (21.25 μg/mL) compared to the XTT test (170 μg/mL). This proves that toxicogenomics is more sensitive, while at the same time being informative about the molecular actions of toxic substances. Interestingly, although the same pathway was activated, there were, *stricto sensu*, no common genes altered by pristine CeO_2_ and NB-DA or NB-DL in the mitochondrial dysfunction pathway as reported in Table 2. Consequently, here, we demonstrated the interest of a pathway-driven analysis compared to the usual gene-driven analysis. In other words, looking at the whole picture is more informative than looking only at key genes.

In addition, these results were perfectly consistent with data obtained by D.A. Pelletier at al., [24] who investigated the effects of cerium oxide NPs on bacterial growth and viability in E. coli, using microarray technology. These authors highlighted concomitant low levels of expression of succinate dehydrogenase and cytochrome b terminal oxidase genes. This indicates that cerium NPs alter electron flow and the respiratory chain in mitochondria. P. Rozenkranz et al. also demonstrated that pristine CeO_2_ NPs decreased mitochondrial activity in H4IIE cells [31].

In the case of the partial or total dissolution of the triammonium citrate layer of NB, the CeO_2_ core becomes exposed. A lack of effect of solubilized ammonium citrate can be assumed since this compound is totally metabolized by most cells in the urea (ammonium) and Krebs (citrate) cycles. Owing to the presence of oxygen vacancies on its surface, and to the self-regenerative cycle of its dual oxidation states, CeO_2_ can scavenge ROS in biological systems. Indeed, when the ratio Ce^3+^/Ce^4+^ falls, the scavenging capabilities increase [32]. Through this property, altered NB adsorbed onto the cell membrane may interact with molecules such as membrane receptors, as suggested by Lee et al. [26], or other molecules, to trigger cell responses via catalytic properties of bare CeO_2_. This can only be a low level response because such interactions likely occur only at nanosized spots.

We showed that the properties of residues of Nanobyk™ nanoparticles tend to be similar to those of pristine CeO_2_ nanoparticles, and different from unaltered Nanobyk™. Consequently, cells exposed to light- or acid-degraded Nanobyk™ were exposed to nanoparticles having surface properties that were closer to those of pristine nanoparticles. The external layer had an efficient protective effect towards toxicity, as we recently observed elsewhere for engineered titanium dioxide NPs including an aluminum oxide protective layer [28]. However, when this layer was altered, the toxic effects, visible by toxicogenomics, increased. Acid alteration of nanoparticles was more severe than light alteration, with regards to the respiratory chain. Macroscopic measurements revealed that all of these NPs were aggregated with identical zeta potential in the culture medium. Thus, the surface properties are important parameters for the toxicity of nanoparticles, whatever their aggregation state. This study examined the acute effects of engineered cerium oxide nanoparticles, which were very moderate. However, the question remains as to their long-term effects: this is another issue to be addressed.

## Conclusion

The capping layer of Nanobyk 3810™ used in outdoor paints has an efficient protective effect against toxicity for intestinal cells. The alteration of this layer, by light or acidic treatment, causes some toxic effects similar to those induced by pristine CeO_2_ nanoparticles. Using toxicogenomics, we found a modification of cell metabolism, especially an alteration of mitochondrial function by underexpression of essential enzymes of the cellular respiratory chain, caused by these altered nanoparticles. The modification of engineered nanomaterials by the environment may increase their toxicity. However, the conclusions regarding cellular toxicity should not be confused with the conclusions on the risk due to these NPs. If the toxicity observed in Caco-2 cells with degraded nanoparticles is slightly higher than that observed with the initial, coated nanoparticles, the risk for humans (dose x exposure) is clearly not increased, as the highest concentrations to which humans may be exposed are currently 100,000 times lower than the concentrations tested [4]. Nevertheless, special care should be taken in designing new nanoparticles, so that they do not transform into more toxic compounds. It can be assumed that a safer design of nanoparticles might include robust protective layers conferring on them more resistance to alterations during their life cycle.

## Methods

### Degradation protocols

Two protocols for NP alteration of the Nanobyk™ (BYK Company) were used in this study. These conditions reproduce extreme and long-term environmental conditions of aging (100% hygrometry and permanent sunshine). Before and after this process, a sample of the suspension was centrifuged (200,000 g) and freeze-dried for fine structural analysis [21]. A Nanobyk™ stock suspension was diluted in milliQ water at 220 mg/mL. Firstly, 500 mL of this suspension was irradiated under artificial daylight for 112 days using OSRAM HQI-BT lamps (E40, 400 W) with a uniform spectral intensity between 425 and 650 nm, and under continuous stirring (100 rpm). A 4 mg/mL stable stock suspension of light-degraded Nanobyk™ (NB-DL) was obtained after 4 months, the time necessary to reach a stable conductivity and pH. This stock suspension of NB-DL was then diluted in a culture medium to obtain the exposure concentrations. Secondly, gastric degradation of the Nanobyk 3810™ was mimicked using a simulated gastric medium (0.2% NaCl, HCl, pH = 1) for 3 h at 37°C [9]. This solution was then neutralized using NaHCO_3_. The stock suspension obtained (NB-DA, 2 mg/mL) was then diluted in the culture medium to obtain the exposure concentrations.

All concentrated suspensions were prepared in culture medium and allowed to be in contact with the medium prior to being diluted to exposure concentrations.

### Physicochemical characterization of CeO_2_-based nanomaterials

The size, shape and mineralogy of Nanobyk™ NPs before and after alteration, as well as of pristine CeO_2_ NPs (originating from Rhodia), were characterized by high-resolution TEM, using a JEOL 2010 F operating at 200 kV. Samples were prepared by evaporating a droplet of the suspension on a carbon-coated copper grid at room temperature. The aggregation states of the nanoparticles were measured by DLS (n = 3) in pure water and in the culture medium, with and without 10% FCS, using the Zetasizer nano ZS (Malvern Instruments Ltd, UK). The zeta potential was also measured on the same instrument. The crystal structure and Ce oxidation state of CeO_2_ NPs and Nanobyk™ NPs were monitored on the atomic scale by X-ray absorption spectroscopy at the Ce L3-edge (5723 eV). Experiments were carried out in transmission mode on the ELETTRA synchrotron (Trieste, Italy) on the XAFS 11.1 beam line [33]. Before and after aging, the suspension was centrifuged (200,000 g) and freeze-dried. The powders were then diluted in PVP and pressed into thin pellets. The spectra were compiled from the merging of three scans, and the energy was calibrated using a standard reference CeO_2_. XANES (X-ray absorption near-edge structure) data were obtained after performing standard procedures for pre-edge subtraction, and normalization using the IFEFFIT software package [34].

### Cell culture and exposure conditions

Caco-2 cells (ATCC, Manassas, VA, US) were cultured in Eagle’s Minimum Essential Medium supplemented with 10% FCS (LGC Standards, Middlesex, UK) and penicillin/streptomycin (100 μg/mL) in a humidified incubator at 37°C, and 5% CO_2_. The cells were used between passages 20 to 40. The cells were passaged weekly at a seeding concentration of 6x10^3^ cells/cm^2^ and the medium was changed three times per week.

### Measurement of transepithelial resistance (TEER)

For TEER measurements, Caco-2 cells were seeded at a density of 5×10^4^ cells per well in 12 Transwell culture plate inserts. MEM supplemented with 10% FCS was added to the apical and basolateral chambers and replenished three times a week. Cultures were confluent at 4 days and stabilized maximum resistance values were reached after 21 days. Transepithelial specific resistance was measured at 37°C using an STX2 electrode with an EvomX recorder (WPI). Blanks (inserts without cells but containing medium) were used to determine baseline values of electrical resistance. Results were expressed in ohms.cm^2^. Each experiment was repeated three times and three measurements were made for each culture plate insert.

### Cytotoxicity tests

#### ATP test

Caco-2 cells were grown in 96-well plates and differentiated for 21 days. The cells were exposed for 24 h or 72 h to serially diluted concentrations of CeO_2_ NPs (21.25, 42.5, 85, and 170 μg/mL, 100 μL per well). Cells were washed with PBS and cell viability was determined by the ATP test as specified by the supplier (CellTiter-Glo luminescent cell viability Assay, Promega). Briefly, 100 μL kit reagent were added per well and the plate was shaken for 10 min at RT before measuring bioluminescence (LUMIstar Galaxy, BMG). Hydrogen peroxide (2.5 mM, 1.25 mM, and 0.625 mM) was used as a positive control.

#### XTT test

Caco-2 cells were grown in 96-well plates and differentiated for 21 days. The cells were exposed for 24 h or 72 h to serially diluted concentrations of CeO_2_ NPs (21.25 to 170 μg/mL, 100 μL per well). Cells were washed with PBS and cell viability was determined by the XTT test as specified by the supplier (In Vitro toxicology assay kit XTT based, Sigma-Aldrich). Briefly, 20 μL kit reagent were added per well and the plate was incubated for 2 h at 37°C before reading absorbance at 450 nm and 690 nm (Multiscan Spectrum, Thermo Electron Corporation). Hydrogen peroxide (2.5 mM, 1.25 mM and 0.625 mM) was used as a positive control.

### Scanning Electron Microscopy (SEM)

Caco-2 cells were seeded at 5×10^4^ cells/cm^2^ in culture medium on clear Millicell-24 Cell Culture Insert Plates with a polyethylene terephthalate (PET) membrane (Millipore) for SEM observation, and allowed to differentiate for 21 days in a 5% CO_2_ incubator. The cells were exposed to highly concentrated NB-type CeO_2_ NPs (170 μg/mL). After 72 h, the cells were washed three times with PBS, fixed with 5% glutaraldehyde in 0.1 M cacodylate for 1 h at 4°C, then washed again twice with distilled water and dehydrated in graded ethanol baths (35, 70, 85, 95 and 100%). Finally, the cells were dehydrated in HMDS (SPI-Chem™) before examination by SEM. The samples were metallized by deposition of carbon (evaporation of braided carbon fiber (Agar scientific Stansted, UK) then analyzed on Balzers MED010 (Balzers Union, Lichtenstein).

### Microarrays and gene expression analysis

For microarray experiments, the cells were seeded in 6-well plates (to collect sufficient amounts of RNA) in the above medium at 5 × 10^4^ cells/cm^2^ and allowed to differentiate for 21 days. The cells were exposed to 21.25 μg/mL for 72 h of all types of CeO_2_ NPs (3 mL/well), in duplicate. The cells were also exposed to hydrogen peroxide for 24 h at 20 μM, as a positive control. Control duplicates were achieved in the vehicle. Each condition of exposure to NPs was compared with a control, i.e. untreated cells (control 1 for NB and NB-DL, control 2 for NB-DA, control 3 for hydrogen peroxide and pristine CeO_2_). The cells were washed extensively to avoid co-extraction of nucleic acids with NPs adsorbed onto the cell surface, collected with trypsin and washed with PBS. The cells were centrifuged and RNA extracted using the RNeasy kit (Qiagen). The RNAs were quantified with the Nanodrop 1000 and their qualities analyzed on an Agilent Bioanalyzer 2100. The RNAs were amplified and labeled with cyanine-3 fluorophore using a QuickAmp kit (Agilent), according to the supplier’s protocol. The efficiency of fluorescent labelling was controlled by UV spectroscopy (Nanodrop 1000) before hybridization on commercial Agilent oligo microarrays (Human V1 4X 44 K) in technical duplicates.

The 44,000 spots represent probes of the whole human genome, including redundancy. The microarrays were scanned with a GenePix 4000B (Axon Instrument Inc., Forster City, CA) in one-color mode at 532 nm and 5 μm resolution. Each condition of exposure to NPs, as well as controls, led to four hybridizations (two biological replicates and two technical replicates). We used Agilent microarrays with four independent genomes per chip (4 × 44,000 probes), thus 8 microarrays are sufficient to perform 32 hybridizations.

In this experimental design, six analyses were conducted: a) control 2 versus control 1, as the experimental negative control, b) hydrogen-peroxide-exposed cells versus unexposed cells (named control 3), as the experimental positive control, and c) surface-untreated (pristine) CeO_2_-NP-exposed cells versus unexposed cells (named control 3), d) NB-exposed cells versus unexposed cells (named control 1), e) NB-DL exposed versus unexposed cells (named control 1) f) NB-DA exposed versus unexposed cells (named control 2). For each analysis, eight raw fluorescence data files were obtained after scanning, which corresponded to four treated cells and four control cells. All files (n = 32) were submitted to GeneSpring software GX11 (Agilent Technologies) for statistical analysis.

Concerning the statistics methodology, we used a widespread method for determining the significance of the change in gene expression [28, 35]. The raw data were first normalized using the percentile shift 75 normalization method. The normalized data were then filtered on the basis of spots present on 100% of the slides in one of two conditions (treated NPs or control). Only spots detected with at least 70% of their pixels above the threshold intensity signal (set to the median background plus two standard deviations) were selected. From the remaining spots, we selected those with fluorescence ratios (representing NP-exposed samples versus unexposed samples) greater than a 1.5-fold-change cut-off, then we determined the statistical significance of the changes with a p-value ≤ 0.05 using a Student’s t-test statistical analysis on Genespring software and performing a Benjamini and Hochberg false discovery rate multiple testing correction. We thus obtained probe sets which were significantly induced or repressed after exposure to various NPs. See Additional file 4: Table S1 in the Supporting Information paragraph.

### Biological analysis

Data were analyzed through the use of IPA (Ingenuity® Systems, http://www.ingenuity.com). Canonical pathways analysis identified the pathways from the IPA library of canonical pathways that were most significant to the data set. Molecules from the dataset that met the fold change cut-off of 1.5 with p-value <0.05, and were associated with a canonical pathway in the Ingenuity Knowledge Base, were considered for the analysis. The significance of the association between the dataset and the canonical pathway was measured in two ways: 1) a ratio of the number of molecules from the dataset that map to the pathway divided by the total number of molecules that map to the canonical pathway is displayed; 2) right-tailed Fisher’s exact test was used to calculate a p-value determining the probability that the association between genes in the dataset and the canonical pathway is explained by chance alone.

### Quantitative RT-PCR

Total RNA was isolated according to the manufacturer’s instructions using the RNeasy kit (Qiagen) and treated with DNase. RNA purity and concentration were determined by UV on a Nanodrop® Spectrophotometer and integrity was assessed on an Agilent 2100 Bioanalyzer (Agilent Technologies). All the samples used in this study showed a 28S/18S ratio indicating intact and pure RNA. Differential analysis of RNA from cells exposed to NPs and unexposed cells was performed by qRT-PCR with the Sybr Green PCR Master Mix (Finzyme) kit, according to the manufacturer’s instructions on Opticon II (Biorad). Primer (Sigma) sequences were, for *ATP5J:* 5′ GTCAGCCGTCTCAGTCCATT 3′ (forward) and 5′ AAAAGCTCCCTCTCCAGCTC 3′ (reverse); for *NDUFA4*: 5′ TCCAGATGTTTGTTGGGACA 3′ (forward) and 5′ GTGGAAAATTGTGCGGATGT 3′ (reverse); for *NDUFS7*: 5′ CGCAAGGTCTACGACCAGAT 3′(forward) and 5′ TCCCGCTTGATCTTCCTCT 3′ (reverse); for *PRDX5:* 5′ GTGGTGGCCTGTCTGAGTGT 3′ (forward) and 5′ ATGCCATCCTGTACCACCAT 3′ (reverse); for *PRDX3:* 5′ GTTGTCGCAGTCTCAGTGGA 3′ (forward) and 5′ GACGCTCAAATGCTTGATGA 3′ (reverse); for *COX6A2*: 5′ CTACCAACACCTCCGCATC 3′ (forward) and 5′ TCGAAGCTTCACACCTTTATTG 3′ (reverse); for *SDHC*: 5′TTGAGTGCAGGGGTCTCTCT 3′ (forward) and 5′ AACCAGGACAACCACTCCAG 3′ (reverse); for *SDHD*: 5′ GTATGCCTCTTTGCCTCTGC 3′ (forward) and 5′ GAGGCAACCCCATTAACTCA 3′ (reverse). For *ATP5J*, *NDUFA4*, *NDUFS7, PRDX5, PRDX3, COX6A2 , SDHC and SDHD*, the amplicon sizes were 186, 199, 241, 203, 216, 155, 240, and 203 bp, respectively. The measurements were the means of three independent experiments, and normalization was based on the total RNA mass quantified on the Nanodrop.

### Availability of supporting data section

The data sets supporting the results of this article are available as additional files (Additional file 4: Table S1).

The raw data discussed in this publication have been deposited in NCBI’s Gene Expression Omnibus (GEO) repository and are accessible through GEO Series accession number GSE60128 (http://www.ncbi.nlm.nih.gov/geo/query/acc.cgi?acc=GSE60128).

## Electronic supplementary material

Additional file 1: Figure S1: Experimental XANES spectra at the Ce L_3_-edge of CeO_2_ NPs, the unaltered Nanobyk™, light-degraded Nanobyk (Nanobyk DL) and acid-degraded Nanobyk (Nanobyk DA), nano-sized CeO_2_, and micron-sized CeO_2_. No change in the cerium redox state was observed between initial and altered Nanobyk. (TIFF 4 MB)

Additional file 2: Figure S2: SEM image of Caco-2 cells exposed for 72 h to 170 μg/mL NPs. Caco-2 cells were grown on bicameral wells (PET, pores 1 μm) and differentiated for 21 days. The cells were exposed to NPs (170 μg/mL). After 72 h incubation, the cells were washed, fixed and dehydrated. They were observed by SEM. Top lane) Magnification 2,000 x. Bottom lane) Magnification 16,000 x. Clear spot deposits are visible at the cell surface only for light-degraded Nanobyk. (TIFF 5 MB)

Additional file 3: Figure S3: Microarray scatter plots. Caco-2 cells were cultured and differentiated for 21 days. They were exposed for 72 h to 21.25 μg/mL CeO_2_ NPs. The scatter plots represent the raw fluorescence intensities of genes filtered at the threshold intensity signal after hybridization (n = 4). From Blue to Red: increasing fluorescence intensity. The number of genes detected above the signal threshold was compared for each type of NP (y-axis) versus their own control (x-axis). A) Unexposed cells versus unexposed cells (control 2 versus control 1) as negative control. B) H_2_O_2_-exposed cells versus unexposed cells (control 3) as positive control. C) Pristine (surface-untreated) cerium-oxide-NP-exposed cells versus unexposed cells (control 3). D) NB-exposed cells versus unexposed cells (control 1). E) NB-DL-exposed cells versus unexposed cells (control 1). F) NB-DA-exposed cells versus unexposed cells (control 2). These graphs do not display the significantly altered genes since they represent the raw fluorescence signals before applying statistical tests. Nevertheless, they give a good, rough overview of the amplitude alterations caused by the different nanoparticles. (TIFF 907 KB)

Additional file 4: Table S1: Complete lists of significant, differentially expressed genes including fold change and p-value in each probe set. NA means no available information. (XLS 2 MB)

Additional file 5: Table S2: List of 37 genes common to NB-DL and NB-DA. (DOC 28 KB)

## Abbreviations

NPs: Nanoparticles
NB: Nanobyk
NB-DL: Nanobyk nanoparticles degraded by light exposure
NB-DA: Nanobyk nanoparticles degraded by acidic treatment with SGF
SGF: Simulated gastric fluid
RT: Room temperature
TEER: Trans-epithelial electric resistance
SEM: Scanning electron microscopy
TEM: Transmission electron microscopy
FCS: Fetal calf serum
PBS: Phosphate-buffered saline
ROS: Reactive oxygen species
HMDS: Hexamethyldisilazane
d: Inter-reticular distance
Dh: Hydrodynamic diameter
PVP: Polyvinylpyrrolidone
EDX: Energy-dispersive X-ray spectroscopy
BSE: Back-scattered electron detector
qRT-PCR: Real-time quantitative polymerase chain reaction.
