# Supplementary figures and images for: Toxicity evaluation of manufactured CeO2 nanoparticles before and after alteration: combined physicochemical and whole-genome expression analysis in Caco-2 cells
Source: BMC Genomics. 2014 Aug 21;15(1):700. doi: 10.1186/1471-2164-15-700 (PMC4150968; doi:10.1186/1471-2164-15-700)

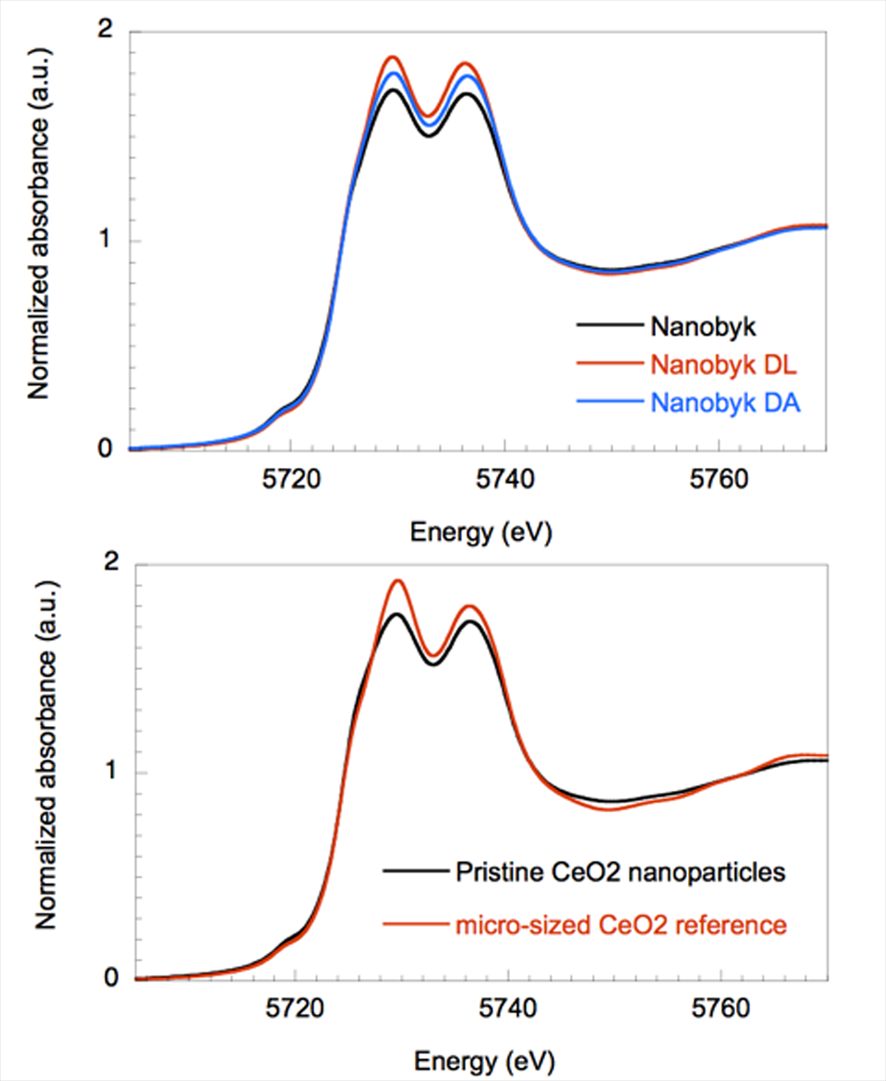

Supplement: Supplementary file 1 — Additional file 1: Figure S1: Experimental XANES spectra at the Ce L3-edge of CeO2 NPs, the unaltered Nanobyk™, light-degraded Nanobyk (Nanobyk DL) and acid-degraded Nanobyk (Nanobyk DA), nano-sized CeO2, and micron-sized CeO2. No change in the cerium redox state was observed between initial and altered Nanobyk. (TIFF 4 MB) [file 12864_2014_6383_MOESM1_ESM.tiff]

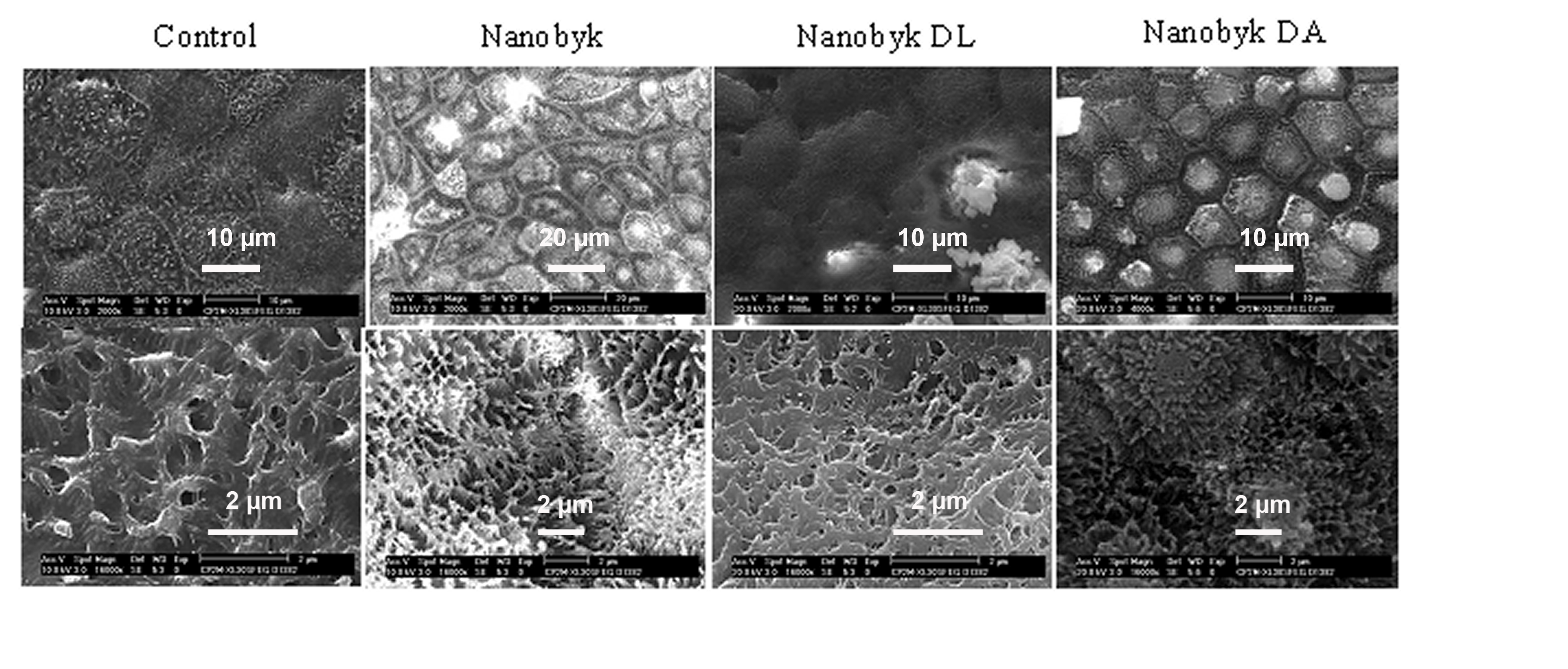

Supplement: Supplementary file 2 — Additional file 2: Figure S2: SEM image of Caco-2 cells exposed for 72 h to 170 μg/mL NPs. Caco-2 cells were grown on bicameral wells (PET, pores 1 μm) and differentiated for 21 days. The cells were exposed to NPs (170 μg/mL). After 72 h incubation, the cells were washed, fixed and dehydrated. They were observed by SEM. Top lane) Magnification 2,000 x. Bottom lane) Magnification 16,000 x. Clear spot deposits are visible at the cell surface only for light-degraded Nanobyk. (TIFF 5 MB) [file 12864_2014_6383_MOESM2_ESM.tiff]

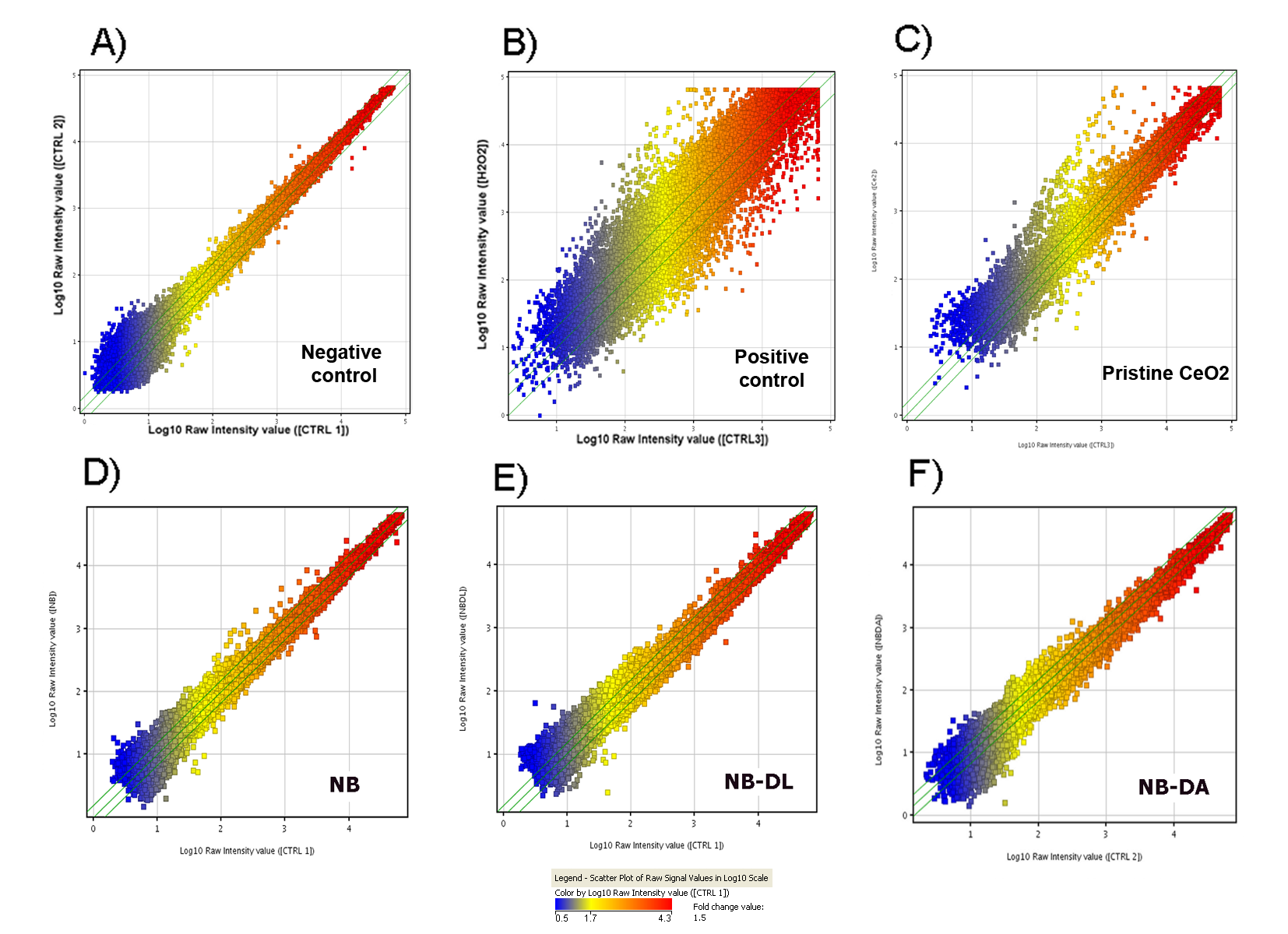

Supplement: Supplementary file 3 — Additional file 3: Figure S3: Microarray scatter plots. Caco-2 cells were cultured and differentiated for 21 days. They were exposed for 72 h to 21.25 μg/mL CeO2 NPs. The scatter plots represent the raw fluorescence intensities of genes filtered at the threshold intensity signal after hybridization (n = 4). From Blue to Red: increasing fluorescence intensity. The number of genes detected above the signal threshold was compared for each type of NP (y-axis) versus their own control (x-axis). A) Unexposed cells versus unexposed cells (control 2 versus control 1) as negative control. B) H2O2-exposed cells versus unexposed cells (control 3) as positive control. C) Pristine (surface-untreated) cerium-oxide-NP-exposed cells versus unexposed cells (control 3). D) NB-exposed cells versus unexposed cells (control 1). E) NB-DL-exposed cells versus unexposed cells (control 1). F) NB-DA-exposed cells versus unexposed cells (control 2). These graphs do not display the significantly altered genes since they represent the raw fluorescence signals before applying statistical tests. Nevertheless, they give a good, rough overview of the amplitude alterations caused by the different nanoparticles. (TIFF 907 KB) [file 12864_2014_6383_MOESM3_ESM.tiff]
