# Supplementary material for: Toxicity evaluation of manufactured CeO2 nanoparticles before and after alteration: combined physicochemical and whole-genome expression analysis in Caco-2 cells
Source: BMC Genomics. 2014 Aug 21;15(1):700. doi: 10.1186/1471-2164-15-700 (PMC4150968; doi:10.1186/1471-2164-15-700)
Supplement: Supplementary file 5 — Additional file 5: Table S2: List of 37 genes common to NB-DL and NB-DA. (DOC 28 KB) [file 12864_2014_6383_MOESM5_ESM.doc]

**Table S2**: 37 differentially expressed genes common to NB-DL and NB-DA treated cells.

Fold changes and p-value are detailed in Table S1for each NPs.
